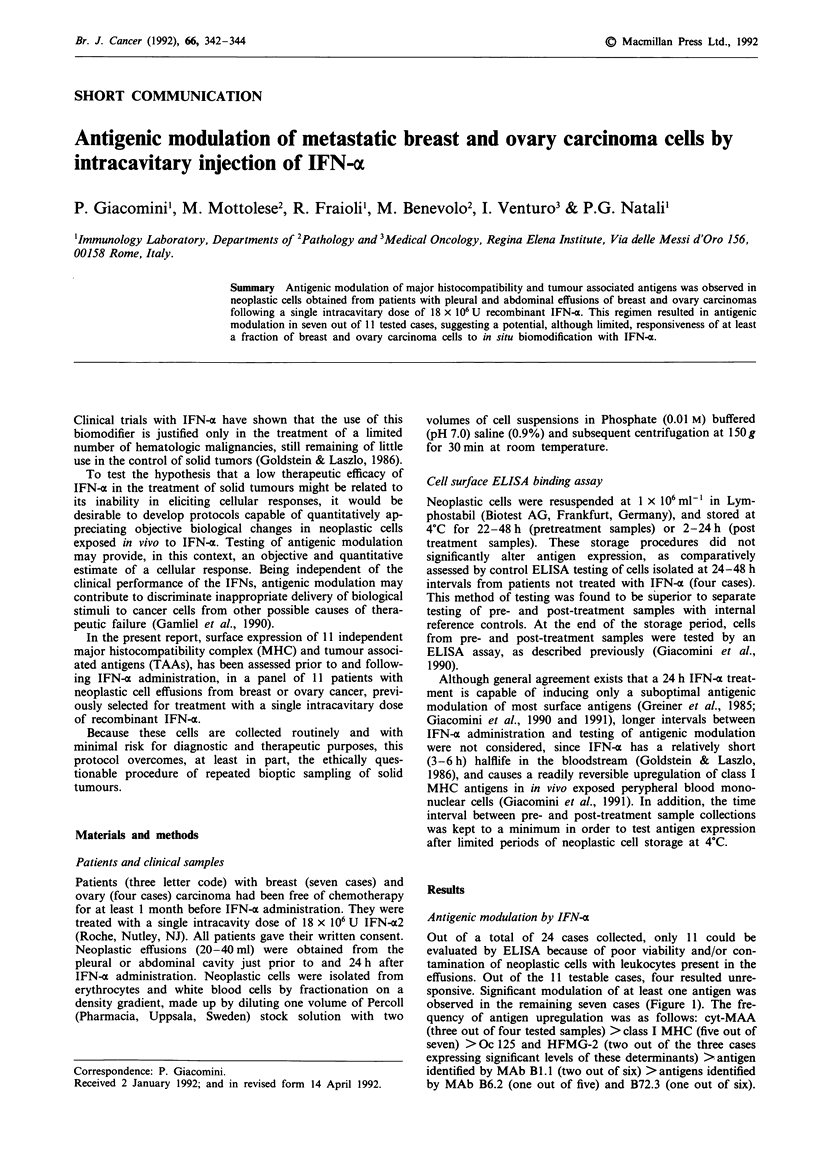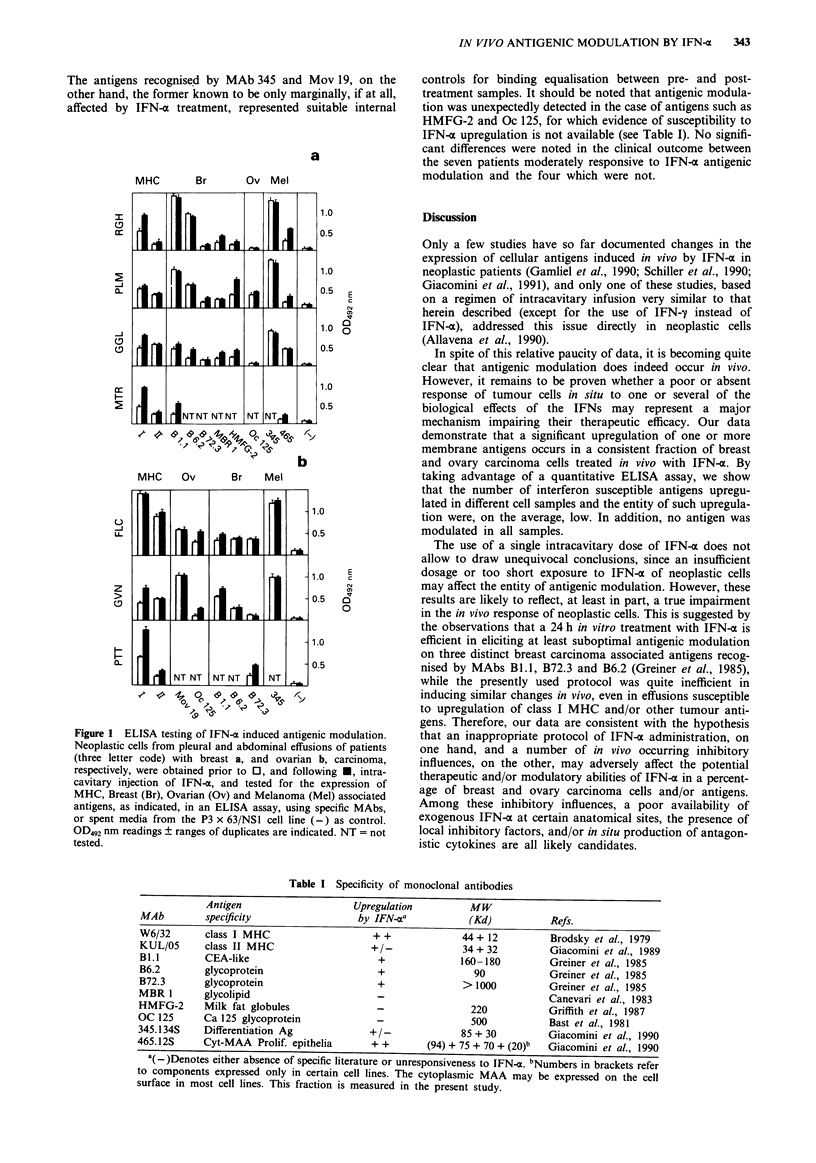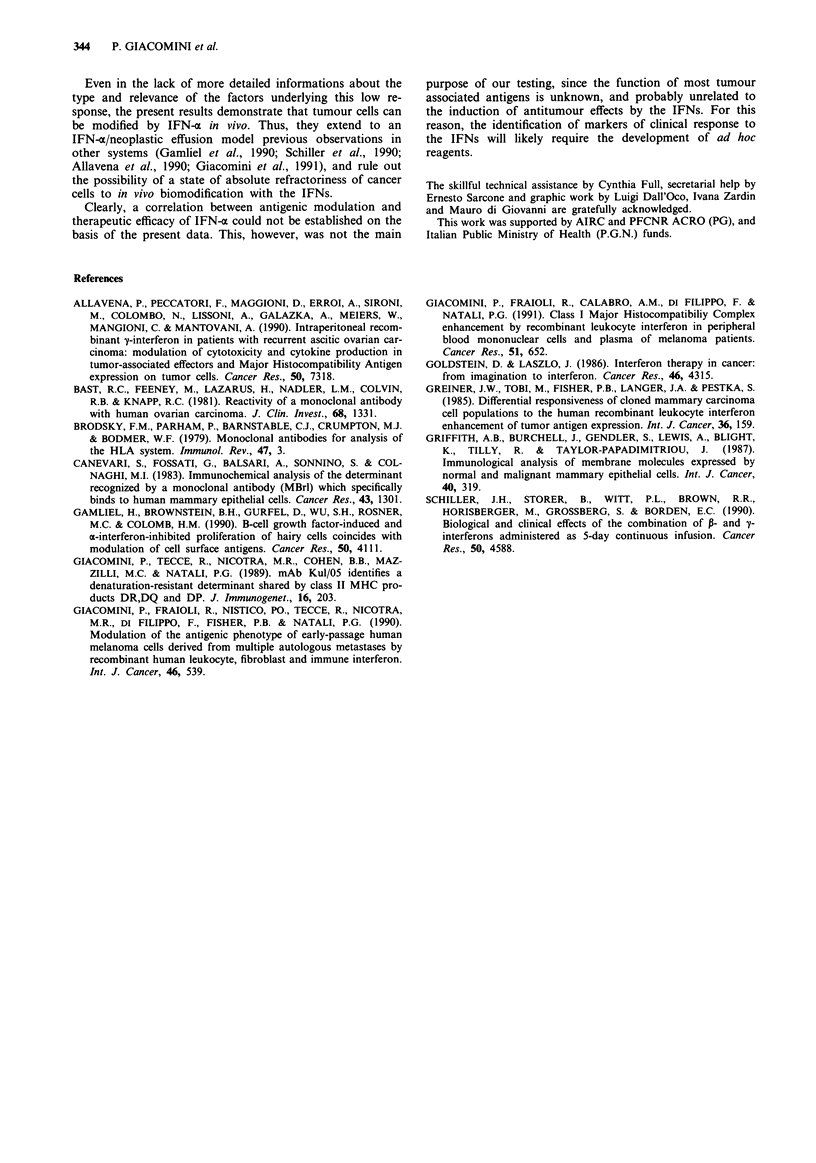# Antigenic modulation of metastatic breast and ovary carcinoma cells by intracavitary injection of IFN-alpha.

**DOI:** 10.1038/bjc.1992.267

**Published:** 1992-08

**Authors:** P. Giacomini, M. Mottolese, R. Fraioli, M. Benevolo, I. Venturo, P. G. Natali

**Affiliations:** Department of Pathology, Regina Elena Institute, Rome, Italy.

## Abstract

Antigenic modulation of major histocompatibility and tumour associated antigens was observed in neoplastic cells obtained from patients with pleural and abdominal effusions of breast and ovary carcinomas following a single intracavitary dose of 18 x 10(6) U recombinant IFN-alpha. This regimen resulted in antigenic modulation in seven out of 11 tested cases, suggesting a potential, although limited, responsiveness of at least a fraction of breast and ovary carcinoma cells to in situ biomodification with IFN-alpha.


*Br. J. Cancer* (1992), **66**, 342–344 © Macmillan Press Ltd., 1992

SHORT COMMUNICATION

### Antigenic modulation of metastatic breast and ovary carcinoma cells by intracavitary injection of IFN-α

P. Giacomini[1], M. Mottolese[2], R. Fraioli[1], M. Benevolo[2], I. Venturo[3] & P.G. Natali[1]

[1]*Immunology Laboratory, Departments of* [2]*Pathology and* [3]*Medical Oncology, Regina Elena Institute, Via delle Messi d'Oro 156, 00158 Rome, Italy.*

**Summary** Antigenic modulation of major histocompatibility and tumour associated antigens was observed in neoplastic cells obtained from patients with pleural and abdominal effusions of breast and ovary carcinomas following a single intracavitary dose of $18 \times 10^6$ U recombinant IFN-α. This regimen resulted in antigenic modulation in seven out of 11 tested cases, suggesting a potential, although limited, responsiveness of at least a fraction of breast and ovary carcinoma cells to *in situ* biomodification with IFN-α.

Clinical trials with IFN-α have shown that the use of this biomodifier is justified only in the treatment of a limited number of hematologic malignancies, still remaining of little use in the control of solid tumors (Goldstein & Laszlo, 1986).

To test the hypothesis that a low therapeutic efficacy of IFN-α in the treatment of solid tumours might be related to its inability in eliciting cellular responses, it would be desirable to develop protocols capable of quantitatively appreciating objective biological changes in neoplastic cells exposed *in vivo* to IFN-α. Testing of antigenic modulation may provide, in this context, an objective and quantitative estimate of a cellular response. Being independent of the clinical performance of the IFNs, antigenic modulation may contribute to discriminate inappropriate delivery of biological stimuli to cancer cells from other possible causes of therapeutic failure (Gamliel *et al.*, 1990).

In the present report, surface expression of 11 independent major histocompatibility complex (MHC) and tumour associated antigens (TAAs), has been assessed prior to and following IFN-α administration, in a panel of 11 patients with neoplastic cell effusions from breast or ovary cancer, previously selected for treatment with a single intracavitary dose of recombinant IFN-α.

Because these cells are collected routinely and with minimal risk for diagnostic and therapeutic purposes, this protocol overcomes, at least in part, the ethically questionable procedure of repeated bioptic sampling of solid tumours.

### Materials and methods

### *Patients and clinical samples*

Patients (three letter code) with breast (seven cases) and ovary (four cases) carcinoma had been free of chemotherapy for at least 1 month before IFN-α administration. They were treated with a single intracavity dose of $18 \times 10^6$ U IFN-α2 (Roche, Nutley, NJ). All patients gave their written consent. Neoplastic effusions (20–40 ml) were obtained from the pleural or abdominal cavity just prior to and 24 h after IFN-α administration. Neoplastic cells were isolated from erythrocytes and white blood cells by fractionation on a density gradient, made up by diluting one volume of Percoll (Pharmacia, Uppsala, Sweden) stock solution with two volumes of cell suspensions in Phosphate (0.01 M) buffered (pH 7.0) saline (0.9%) and subsequent centrifugation at 150 *g* for 30 min at room temperature.

### *Cell surface ELISA binding assay*

Neoplastic cells were resuspended at $1 \times 10^6$ ml$^{-1}$ in Lymphostabil (Biotest AG, Frankfurt, Germany), and stored at 4°C for 22–48 h (pretreatment samples) or 2–24 h (post treatment samples). These storage procedures did not significantly alter antigen expression, as comparatively assessed by control ELISA testing of cells isolated at 24–48 h intervals from patients not treated with IFN-α (four cases). This method of testing was found to be superior to separate testing of pre- and post-treatment samples with internal reference controls. At the end of the storage period, cells from pre- and post-treatment samples were tested by an ELISA assay, as described previously (Giacomini *et al.*, 1990).

Although general agreement exists that a 24 h IFN-α treatment is capable of inducing only a suboptimal antigenic modulation of most surface antigens (Greiner *et al.*, 1985; Giacomini *et al.*, 1990 and 1991), longer intervals between IFN-α administration and testing of antigenic modulation were not considered, since IFN-α has a relatively short (3–6 h) halflife in the bloodstream (Goldstein & Laszlo, 1986), and causes a readily reversible upregulation of class I MHC antigens in *in vivo* exposed perypheral blood mononuclear cells (Giacomini *et al.*, 1991). In addition, the time interval between pre- and post-treatment sample collections was kept to a minimum in order to test antigen expression after limited periods of neoplastic cell storage at 4°C.

### Results

### *Antigenic modulation by IFN-α*

Out of a total of 24 cases collected, only 11 could be evaluated by ELISA because of poor viability and/or contamination of neoplastic cells with leukocytes present in the effusions. Out of the 11 testable cases, four resulted unresponsive. Significant modulation of at least one antigen was observed in the remaining seven cases (Figure 1). The frequency of antigen upregulation was as follows: cyt-MAA (three out of four tested samples) > class I MHC (five out of seven) > Oc 125 and HFMG-2 (two out of the three cases expressing significant levels of these determinants) > antigen identified by MAb B1.1 (two out of six) > antigens identified by MAb B6.2 (one out of five) and B72.3 (one out of six).

Correspondence: P. Giacomini.
Received 2 January 1992; and in revised form 14 April 1992.

The antigens recognised by MAb 345 and Mov 19, on the other hand, the former known to be only marginally, if at all, affected by IFN-α treatment, represented suitable internal controls for binding equalisation between pre- and post-treatment samples. It should be noted that antigenic modulation was unexpectedly detected in the case of antigens such as HMFG-2 and Oc 125, for which evidence of susceptibility to IFN-α upregulation is not available (see Table I). No significant differences were noted in the clinical outcome between the seven patients moderately responsive to IFN-α antigenic modulation and the four which were not.

**Figure 1** ELISA testing of IFN-α induced antigenic modulation. Neoplastic cells from pleural and abdominal effusions of patients (three letter code) with breast **a**, and ovarian **b**, carcinoma, respectively, were obtained prior to □, and following ■, intracavitary injection of IFN-α, and tested for the expression of MHC, Breast (Br), Ovarian (Ov) and Melanoma (Mel) associated antigens, as indicated, in an ELISA assay, using specific MAbs, or spent media from the P3 × 63/NS1 cell line (−) as control. $OD_{492}$ nm readings ± ranges of duplicates are indicated. NT = not tested.

### Discussion

Only a few studies have so far documented changes in the expression of cellular antigens induced *in vivo* by IFN-α in neoplastic patients (Gamliel *et al.*, 1990; Schiller *et al.*, 1990; Giacomini *et al.*, 1991), and only one of these studies, based on a regimen of intracavitary infusion very similar to that herein described (except for the use of IFN-γ instead of IFN-α), addressed this issue directly in neoplastic cells (Allavena *et al.*, 1990).

In spite of this relative paucity of data, it is becoming quite clear that antigenic modulation does indeed occur *in vivo*. However, it remains to be proven whether a poor or absent response of tumour cells *in situ* to one or several of the biological effects of the IFNs may represent a major mechanism impairing their therapeutic efficacy. Our data demonstrate that a significant upregulation of one or more membrane antigens occurs in a consistent fraction of breast and ovary carcinoma cells treated *in vivo* with IFN-α. By taking advantage of a quantitative ELISA assay, we show that the number of interferon susceptible antigens upregulated in different cell samples and the entity of such upregulation were, on the average, low. In addition, no antigen was modulated in all samples.

The use of a single intracavitary dose of IFN-α does not allow to draw unequivocal conclusions, since an insufficient dosage or too short exposure to IFN-α of neoplastic cells may affect the entity of antigenic modulation. However, these results are likely to reflect, at least in part, a true impairment in the *in vivo* response of neoplastic cells. This is suggested by the observations that a 24 h *in vitro* treatment with IFN-α is efficient in eliciting at least suboptimal antigenic modulation on three distinct breast carcinoma associated antigens recognised by MAbs B1.1, B72.3 and B6.2 (Greiner *et al.*, 1985), while the presently used protocol was quite inefficient in inducing similar changes *in vivo*, even in effusions susceptible to upregulation of class I MHC and/or other tumour antigens. Therefore, our data are consistent with the hypothesis that an inappropriate protocol of IFN-α administration, on one hand, and a number of *in vivo* occurring inhibitory influences, on the other, may adversely affect the potential therapeutic and/or modulatory abilities of IFN-α in a percentage of breast and ovary carcinoma cells and/or antigens. Among these inhibitory influences, a poor availability of exogenous IFN-α at certain anatomical sites, the presence of local inhibitory factors, and/or *in situ* production of antagonistic cytokines are all likely candidates.

**Table I** Specificity of monoclonal antibodies

| *MAb* | *Antigen specificity* | *Upregulation by IFN-α*[a] | *MW (Kd)* | *Refs.* |
|---|---|---|---|---|
| W6/32 | class I MHC | + + | 44 + 12 | Brodsky *et al.*, 1979 |
| KUL/05 | class II MHC | +/− | 34 + 32 | Giacomini *et al.*, 1989 |
| B1.1 | CEA-like | + | 160–180 | Greiner *et al.*, 1985 |
| B6.2 | glycoprotein | + | 90 | Greiner *et al.*, 1985 |
| B72.3 | glycoprotein | + | > 1000 | Greiner *et al.*, 1985 |
| MBR 1 | glycolipid | − | | Canevari *et al.*, 1983 |
| HMFG-2 | Milk fat globules | − | 220 | Griffith *et al.*, 1987 |
| OC 125 | Ca 125 glycoprotein | − | 500 | Bast *et al.*, 1981 |
| 345.134S | Differentiation Ag | +/− | 85 + 30 | Giacomini *et al.*, 1990 |
| 465.12S | Cyt-MAA Prolif. epithelia | + + | (94) + 75 + 70 + (20)[b] | Giacomini *et al.*, 1990 |

[a](−)Denotes either absence of specific literature or unresponsiveness to IFN-α. [b]Numbers in brackets refer to components expressed only in certain cell lines. The cytoplasmic MAA may be expressed on the cell surface in most cell lines. This fraction is measured in the present study.

Even in the lack of more detailed informations about the type and relevance of the factors underlying this low response, the present results demonstrate that tumour cells can be modified by IFN-α *in vivo*. Thus, they extend to an IFN-α/neoplastic effusion model previous observations in other systems (Gamliel *et al.*, 1990; Schiller *et al.*, 1990; Allavena *et al.*, 1990; Giacomini *et al.*, 1991), and rule out the possibility of a state of absolute refractoriness of cancer cells to *in vivo* biomodification with the IFNs.

Clearly, a correlation between antigenic modulation and therapeutic efficacy of IFN-α could not be established on the basis of the present data. This, however, was not the main purpose of our testing, since the function of most tumour associated antigens is unknown, and probably unrelated to the induction of antitumour effects by the IFNs. For this reason, the identification of markers of clinical response to the IFNs will likely require the development of *ad hoc* reagents.

The skillful technical assistance by Cynthia Full, secretarial help by Ernesto Sarcone and graphic work by Luigi Dall'Oco, Ivana Zardin and Mauro di Giovanni are gratefully acknowledged.

This work was supported by AIRC and PFCNR ACRO (PG), and Italian Public Ministry of Health (P.G.N.) funds.

### References

ALLAVENA, P., PECCATORI, F., MAGGIONI, D., ERROI, A., SIRONI, M., COLOMBO, N., LISSONI, A., GALAZKA, A., MEIERS, W., MANGIONI, C. & MANTOVANI, A. (1990). Intraperitoneal recombinant γ-interferon in patients with recurrent ascitic ovarian carcinoma: modulation of cytotoxicity and cytokine production in tumor-associated effectors and Major Histocompatibility Antigen expression on tumor cells. *Cancer Res.*, **50**, 7318.

BAST, R.C., FEENEY, M., LAZARUS, H., NADLER, L.M., COLVIN, R.B. & KNAPP, R.C. (1981). Reactivity of a monoclonal antibody with human ovarian carcinoma. *J. Clin. Invest.*, **68**, 1331.

BRODSKY, F.M., PARHAM, P., BARNSTABLE, C.J., CRUMPTON, M.J. & BODMER, W.F. (1979). Monoclonal antibodies for analysis of the HLA system. *Immunol. Rev.*, **47**, 3.

CANEVARI, S., FOSSATI, G., BALSARI, A., SONNINO, S. & COLNAGHI, M.I. (1983). Immunochemical analysis of the determinant recognized by a monoclonal antibody (MBrl) which specifically binds to human mammary epithelial cells. *Cancer Res.*, **43**, 1301.

GAMLIEL, H., BROWNSTEIN, B.H., GURFEL, D., WU, S.H., ROSNER, M.C. & COLOMB, H.M. (1990). B-cell growth factor-induced and α-interferon-inhibited proliferation of hairy cells coincides with modulation of cell surface antigens. *Cancer Res.*, **50**, 4111.

GIACOMINI, P., TECCE, R., NICOTRA, M.R., COHEN, B.B., MAZZILLI, M.C. & NATALI, P.G. (1989). mAb Kul/05 identifies a denaturation-resistant determinant shared by class II MHC products DR,DQ and DP. *J. Immunogenet.*, **16**, 203.

GIACOMINI, P., FRAIOLI, R., NISTICO, PO., TECCE, R., NICOTRA, M.R., DI FILIPPO, F., FISHER, P.B. & NATALI, P.G. (1990). Modulation of the antigenic phenotype of early-passage human melanoma cells derived from multiple autologous metastases by recombinant human leukocyte, fibroblast and immune interferon. *Int. J. Cancer*, **46**, 539.

GIACOMINI, P., FRAIOLI, R., CALABRO, A.M., DI FILIPPO, F. & NATALI, P.G. (1991). Class I Major Histocompatibiliy Complex enhancement by recombinant leukocyte interferon in peripheral blood mononuclear cells and plasma of melanoma patients. *Cancer Res.*, **51**, 652.

GOLDSTEIN, D. & LASZLO, J. (1986). Interferon therapy in cancer: from imagination to interferon. *Cancer Res.*, **46**, 4315.

GREINER, J.W., TOBI, M., FISHER, P.B., LANGER, J.A. & PESTKA, S. (1985). Differential responsiveness of cloned mammary carcinoma cell populations to the human recombinant leukocyte interferon enhancement of tumor antigen expression. *Int. J. Cancer*, **36**, 159.

GRIFFITH, A.B., BURCHELL, J., GENDLER, S., LEWIS, A., BLIGHT, K., TILLY, R. & TAYLOR-PAPADIMITRIOU, J. (1987). Immunological analysis of membrane molecules expressed by normal and malignant mammary epithelial cells. *Int. J. Cancer*, **40**, 319.

SCHILLER, J.H., STORER, B., WITT, P.L., BROWN, R.R., HORISBERGER, M., GROSSBERG, S. & BORDEN, E.C. (1990). Biological and clinical effects of the combination of β- and γ-interferons administered as 5-day continuous infusion. *Cancer Res.*, **50**, 4588.